# Adaptation and latent structure of the Brazilian version of the Ego Dissolution Inventory (EDI-BR): an exploratory study

**DOI:** 10.47626/2237-6089-2022-0491

**Published:** 2024-04-23

**Authors:** Bheatrix Bienemann, Marcio S. C. Longo, Mariana Ridolfi, Marco Multedo, Lucas V. M. Cruz, Eduardo Schenberg, Luis Fernando Tófoli, Daniel C. Mograbi

**Affiliations:** 1 Departamento de Psicologia Pontifícia Universidade Católica do Rio de Janeiro Rio de Janeiro RJ Brazil Departamento de Psicologia, Pontifícia Universidade Católica do Rio de Janeiro, Rio de Janeiro, RJ, Brazil.; 2 Instituto de Psiquiatria Universidade Federal do Rio de Janeiro Rio de Janeiro RJ Brazil Instituto de Psiquiatria, Universidade Federal do Rio de Janeiro, Rio de Janeiro, RJ, Brazil.; 3 Instituto Phaneros São Paulo SP Brazil Instituto Phaneros, São Paulo, SP, Brazil.; 4 Cooperação Interdisciplinar para Pesquisa e Divulgação da Ayahuasca Faculdade de Ciências Médicas Universidade Estadual de Campinas Campinas SP Brazil Cooperação Interdisciplinar para Pesquisa e Divulgação da Ayahuasca, Faculdade de Ciências Médicas, Universidade Estadual de Campinas, Campinas, SP, Brazil.; 5 Institute of Psychiatry, Psychology & Neuroscience King’s College London London UK Institute of Psychiatry, Psychology & Neuroscience, King’s College London, London, UK.

**Keywords:** Ego dissolution, ego death, psychedelics, hallucinogens, psychometrics

## Abstract

**Objective:**

Existing scales that seek to measure alterations in self-experience were developed based on studies conducted in developed countries. Therefore, the aim of this study was to evaluate the psychometric properties of the Ego Dissolution Inventory (EDI) after translating and adapting it for the Brazilian context.

**Methods:**

The measure was translated by two translators fluent in both English and Portuguese, followed by back-translation into English to ensure there was no loss of meaning. The scale was used in an online survey exploring substance use. A total of 528 participants answered the full scale. We calculated the Kaiser-Meyer-Olkin (KMO) measure to evaluate sampling adequacy, then ran exploratory factor analysis (EFAs) to investigate the factor structure of the EDI.

**Results:**

The scale showed acceptable psychometric properties, with excellent internal consistency and sampling adequacy for factor analysis. Kaiser-Guttman’s criteria and Hull’s method indicated a three-factor solution, while parallel analysis suggested a two-factor solution. All items showed salient loadings, with two items exhibiting cross-loading. Positive but weak correlations were found between EDI factors 1 and 2 and nature relatedness.

**Conclusions:**

The validated scale showed solid psychometric properties, with potential differences in factor structure in relation to the English version. Considering validation is an ongoing process, it is recommended that studies be conducted comparing ego dissolution scores across distinct substances and different regions of the country.

## Introduction

Psychedelics are a class of substance that produce transient but intense changes in perception, mood, and feelings.^1^ These substances act mainly on serotoninergic systems, through 5-HT_2A_ receptor affinity.^2^ Several studies suggest the therapeutic potential of these substances, in cases such as supportive care for anxiety in terminal patients,^3^ treatment of refractory depression,^4^ and management of substance use disorders.^5^

Ego dissolution (also called “ego death”)^6^ is a central feature of the psychedelic experience and a promising topic in the study of consciousness. It is characterized as a sensation of blurred distinction between the subjective and objective perception of the world,^7^ and feelings of connectedness with the world.^8^ Reported effects of drug-induced ego dissolution studies include feeling of numbness, feelings of “non-existence,” disembodiment, depersonalization, and feelings of unity with the environment.^9^ This phenomenon can occur in some pathological states, such as in acute psychosis or in temporal lobe epilepsy auras,^10^ but also in states considered non-pathological, such as in mystical experiences or through altered states of consciousness induced by hallucinogenic substances.^7^

There are several instruments that seek to measure alterations in self-experience. Dittrich’s APZ (Abnormal Mental States) questionnaire^11^ as well as its revised versions the Five Dimensional Altered States of Consciousness (5D-ASC) and the Psychometric Evaluation of the Altered States of Consciousness Rating Scale (OAV)^12^ have been widely used to assess altered states of consciousness caused by psychedelic substances. However, the large number of items and the complex 11-factor structure is seen as a limitation by researchers.^7^ For this reason, Nour et al.^7^ sought to develop a measure with a simpler one-dimensional structure: the Ego dissolution Inventory (EDI). However, these measures all originated in developed countries. To the best of our knowledge, there are no studies conducted in developing regions to validate scales measuring ego dissolution. Considering the traditional use of psychedelics in these cultures (e.g., psilocybe mushrooms, ayahuasca, peyote), the absence of adequately validated measures for this population constitutes an important gap in psychedelic science.

Additionally, ego dissolution may have important clinical implications. For example, in a study by Uthaug et al.^13^ evaluating long-term effects of ayahuasca use, participants’ ego dissolution during psychedelic experiences was strongly linked to decreases in depressive symptoms and increased life satisfaction. This suggests that ego dissolution is a relevant element involved in the therapeutic action of psychedelics. On the other hand, there is also evidence of negative outcomes in relation to the phenomenon of ego dissolution, such as bad trips, anxiety, and seeking emergency medical care.^14^ Therefore, it is important that clinical studies with psychedelics seek to measure this construct for a greater understanding of its relationship with positive and negative outcomes. Accordingly, the aim of the current study was to translate and adapt the EDI for the Brazilian context, evaluating its psychometric properties.

Although the final version of the EDI comprises eight items referring to the phenomenon of ego dissolution, the original scale included eight additional items, tapping into the (presumably opposite) phenomenon of ego inflation.^7^ Assuming that inflation and dissolution are indeed contrary phenomena, but still along the spectrum of the same construct, we chose to use the original 16-item scale and evaluate its psychometric structure in a sample from a developing country. It was expected that, as in the original study, the items would load onto two different factors: ego dissolution and ego inflation.

## Methods

### Scale adaptation

Cultural adaptation was conducted following procedures that are standard in the field,^15^ with translation into Brazilian Portuguese by two translators fluent in both English and Portuguese. A back-translation into English was performed by a third translator, also fluent in both English and Portuguese, and then compared with the original scale to determine whether there were any changes in the meaning of items. The final scale items were also evaluated by a group of three experts and a pilot/pre-test study was carried out with 11 people before the official testing. There were no major changes in the meaning of items. The scale was included in an online survey exploring substance use, implemented and hosted on SurveyMonkey.^16^ The full survey took an average of 25 minutes to complete and was disseminated online, through social networks and mailing lists.

### Participants

The sample comprised 528 individuals. Respondents were preponderantly women (59.1%) with an average age of 29 years. The majority (80%) declared themselves as white, 44.2% had a bachelor’s degree or higher, and 30.9% had only attended elementary or high school. Regarding their region of residence in Brazil, the vast majority (68.4%) were from the Southeast, with the largest contingent of respondents being from the state of Rio de Janeiro (n = 178), followed by São Paulo (n = 159), and Minas Gerais (n = 21). Inclusion criteria for participants were: (1) being at least 18 years old; and (2) having had at least one experience with a classical psychedelic (LSD, psilocybin, DMT, or ayahuasca), MDMA, cocaine, marijuana, and/or alcohol. The complete sociodemographic data for the sample can be consulted in Table 1.

**Table 1:** Sociodemographic characteristics of the sample

Variables	n (%)
Sex	
Female	312 (59.1)
Male	210 (39.8)
Other	6 (1.1)
Sexual orientation	
Heterosexual	333 (63.1)
Bisexual	138 (26.1)
Gay/lesbian	45 (8.5)
Other	12 (2.3)
Ethnicity/race (mv = 9)	
White/Caucasian	422 (80.0)
Afro-descendant	90 (17.0)
Other	16 (3.0)
Region	
South	46 (8.7)
Southeast	361 (68.4)
Midwest	12 (2.3)
Northeast	26 (5.0)
North	0 (0.0)
Educational level (mv = 116)	
Elementary school/high school	163 (30.9)
University education	167 (31.7)
Graduate education	66 (12.5)
Other	16 (3.0)
Marital status	
Single	367 (69.5)
Married/civil union	128 (24.2)
Divorced/widowed/other	33 (6.2)
Substance used	
Psilocybin mushrooms	145 (27.6)
LSD	162 (30.7)
DMT	47 (8.9)
MDMA	53 (10.0)
Cocaine	16 (3.0)
Cannabis	43 (8.1)
Alcohol	62 (11.7)
Age*	29.0 (9.7)

mv = missing values.

* Mean (standard deviation).

### Instruments

#### Sociodemographic questionnaire

Sociodemographic data relevant to the research, such as gender, age, education, course, income, marital status, and other variables, were collected from the participants.

#### Substance use questionnaire

This questionnaire was designed with the aim of mapping substance use among participants, contexts in which use occurs, and ways of obtaining substances, as well as exploring possible problems and negative outcomes associated with substance use as part of a major study.

In addition to the EDI, the instruments described were also used to explore convergent validity.

*Brief measure of Nature Relatedness Scale (NR6)^17^ (Brazilian version by Longo et al., “Brazilian adaptation and validation of the short-form version of the Nature Relatedness Scale – NR-6,” personal communication).*

This instrument is designed to assess subjects’ relationship with nature, a construct that has become increasingly useful in the study of environmental behavior, as well as health and psychological well-being.^17^ Recent research indicates that nature relatedness increases after psychedelic use,^18-21^ which may be mediated by ego loss phenomenon and the sensation of connectedness with the whole.^19^ This is a one-dimensional, six-item scale with a five-point Likert-type response scale, with higher scores corresponding to higher levels of nature relatedness. The original version of the NR6 has a mean Cronbach’s alpha (α) of 0.84, with the Brazilian version showing similar internal consistency (α = 0.86) and factor structure (Longo et al., personal communication).

#### Satisfaction with Life Scale (SWLS)^22^ (Brazilian version by Gouveia et al.^23^)

This is a one-dimensional Likert-type scale, with higher scores corresponding to higher levels of satisfaction with life. There is evidence that use of psychedelics increases levels of life satisfaction^24^ and it appears that ego dissolution is significantly related to this specific improvement.^7,13,25^ The scale has solid psychometric properties (α = 0.87 for the original scale,^22^ α = 0.81 for Brazilian version^23^).

## Statistics

The Kaiser-Meyer-Olkin (KMO) measure was calculated to evaluate sampling adequacy for factor analysis. It has been suggested that KMO values should be equal to or above 0.60 in order to perform and satisfactorily interpret a factor analysis solution.^26^ Exploratory factor analyses (EFAs) were calculated to investigate the factor structure of the EDI-BR. Principal axis factoring was used as the extraction method, with Promax factor rotation,^27^ an oblique method suited for data in which the factors are potentially correlated.^28^ Kaiser-Guttman’s criteria (eigenvalue > 1), parallel analysis,^29^ and Hull’s method^30^ were each used as the factor retention method in three different analyses. Cronbach’s alpha was calculated for the full scale as well as for individual factors. Correlation analyses were calculated between all scale factors and scores in the SWLS and NR6 scales. The analyses were conducted with factor 10.10.01^31^ and IBM SPSS Statistics v.23.^32^

## Ethical considerations

This study was approved by a local research ethics committee (CAAE: 95292418.5.0000.8144). All participants provided informed consent before completing the questionnaires.

## Results

The KMO index indicated very good sampling adequacy (KMO = 0.905; Bartlett’s test p < 0.001), suggesting that the correlation matrix was suitable for factor analysis. Cronbach’s alpha was 0.95 for the full scale.

### Exploratory factor analysis

Both Kaiser-Guttman’s criteria and Hull’s method indicate a three-factor solution, while parallel analysis suggested a two-factor solution. Table 2 shows results for the three-factor solution using Kaiser-Guttman’s criteria, while Table 3 lists results for the two-factor solution.

**Table 2:** Factor loadings of the EDI scale items obtained with principal axis factoring analysis and Promax rotation

Item #	Item	EDI factor 1	EDI factor 2	EDI factor 3
14	I felt particularly self-confident	**0.91**	-0.16	0.09
16	I felt particularly safe	**0.84**	-0.05	-0.08
8	I felt particularly sure-of-myself	**0.81**	-0.04	0.03
5	I felt a sense of union with others	**0.66**	0.23	-0.12
2	I felt particularly assertive	**0.61**	0.15	0.07
11	I felt far less absorbed by my own issues and concerns	**0.51**	0.10	0.03
10	I felt especially motivated and competitive	**0.47**	-0.11	0.31
9	I experienced a disintegration of my “self” or ego	-0.04	**0.90**	0.05
15	All notion of self and identity dissolved	-0.12	**0.83**	0.08
13	I lost all sense of ego	0.02	**0.79**	-0.02
1	I experienced a dissolution of my “self” or ego	0.11	**0.78**	-0.01
7	I experienced a decrease in my sense of self-importance	-0.08	**0.58**	0.07
3	I felt at one with the universe	0.40	**0.52**	-0.13
12	I felt as if my viewpoint was worth more than other peoples’	-0.10	0.09	**0.79**
4	I felt more important or special than others	0.02	0.11	**0.70**
6	My ego felt inflated	0.17	-0.08	**0.65**
				
Eigenvalue		5.67	2.59	1.42
Variance (%)		35.46	16.16	8.89
Cronbach’s alpha		0.81	0.90	0.71
Root mean square error of approximation (RMSEA)		0.084		
Comparative fit index (CFI)		0.955		
Schwarz’s Bayesian information criterion (BIC)		757.679		

EDI = Ego Dissolution Inventory.

Factor loadings greater than 0.4 are shown in bold.

**Table 3:** Factor loadings of the EDI scale items obtained with parallel analysis and Promax rotation

Item #	Item	EDI factor 1	EDI factor 2
1	I experienced a dissolution of my “self” or ego	**0.86**	0.01
3	I felt at one with the universe	**0.68**	0.18
5	I felt a sense of union with others	**0.45**	**0.40**
7	I experienced a decrease in my sense of self-importance	**0.63**	-0.10
9	I experienced a disintegration of my “self” or ego	**0.94**	-0.12
13	I lost all sense of ego	**0.90**	-0.14
15	All notion of self and identity dissolved	**0.87**	-0.12
2	I felt particularly assertive	0.32	**0.55**
4	I felt more important or special than others	-0.11	**0.67**
6	My ego felt inflated	-0.24	**0.73**
8	I felt particularly sure-of-myself	0.16	**0.70**
10	I felt especially motivated and competitive	-0.11	**0.68**
11	I felt far less absorbed by my own issues and concerns	0.21	**0.43**
12	I felt as if my viewpoint was worth more than other peoples’	-0.17	**0.65**
14	I felt particularly self-confident	0.01	**0.83**
16	I felt particularly safe	0.14	**0.65**
			
Eigenvalue		6.22	1.77
Variance (%)		45.32	15.14
Cronbach’s Alpha		0.89	0.81
Root mean square error of approximation (RMSEA)		0.119	
Comparative fit index (CFI)		0.934	
Schwarz’s Bayesian information criterion (BIC)		1048.584	

EDI = Ego Dissolution Inventory.

Factor loadings greater than 0.4 are represented in bold

#### Correlations between EDI factors, NR6, and SWLS

Results can be seen in Table 4. All EDI factors correlated positively. Ego dissolution did not correlate with SWLS scores. EDI factors 1 and 2 correlated weakly with NR6 scores.

**Table 4:** Correlations between the EDI, NR6, and SWLS

Spearman’s rho	EDI factor 1	EDI factor 2	EDI factor 3
EDI factor 1		0.58*	0.47*
EDI factor 2			0.35*
NR6	0.22*	0.30*	0.01
SWLS	0.05	0.01	-0.07

EDI = Ego dissolution Inventory; NR6 = Nature Relatedness Scale; SWLS = Satisfaction with Life.

* p < 0.01.

## Ego dissolution and substance use

One-way analysis of variance (ANOVA) was also used to compare EDI scores for each factor of the three-factor solution between users of different substances. There were significant differences for all EDI factors (factor 1: *F*[6, 521] = 12.30; p < 0.001; *η*^2^ = 0.12/factor 2: *F*[6, 521] = 25.72; p < 0.001; *η*^2^ = 0.23/factor 3: *F*[6, 520] = 6.52; p < 0.001; *η*^2^ = 0.07).

Full results with all pairwise comparisons for each factor can be consulted in Tables S1, S2, and S3, available as online-only supplementary material. Post-hoc Bonferroni corrected *t* tests showed that mushroom, DMT, LSD, and MDMA users had higher scores than cannabis and alcohol users in factor 1 (“self-confidence and assertiveness”). LSD users also scored significantly higher on this factor than MDMA users, with cocaine users also scoring higher than cannabis users. For factor 2 (“ego dissolution”), both mushroom and DMT users scored significantly higher than users of all other substances. DMT users also scored significantly higher than mushroom users, and LSD users score higher than cannabis and alcohol users. For factor 3 (“ego inflation”), MDMA and cocaine users scored significantly higher than magic mushroom, DMT, and cannabis users. Cocaine users also scored higher than LSD and alcohol users.

## Discussion

The validated version of the scale showed acceptable psychometric properties, with excellent internal consistency for the full scale (and excellent to satisfactory consistency for individual factors) and salient loadings for all items. Factor analyses of the EDI-BR indicated different solutions. Analyses based on the Kaiser-Guttman criteria and Hull method indicated that a three-factor solution was a better fit to the data, while parallel analysis suggested a two-factor solution. All EDI factors correlated positively and there were weak positive correlations between ego dissolution and nature relatedness. The final version of the scale can be consulted in the supplementary material S4.

The authors of the original validation article^7^ included eight items related to ego dissolution and eight items related to ego inflation. This structure was corroborated by the exploratory factor analysis performed, with retention of factors through parallel analysis. In the present study, although the parallel analysis also pointed to a two-factor solution, the items did not behave in the same way. Items specifically referring to the feeling of ego disintegration (e.g., items 1, 3, 5, 7, 9, 13, and 15) grouped onto one factor, while other items grouped onto a second factor.

However, in this two-factor model, item 5 (“I felt union with others”) showed cross-loadings (factor 1: 0.45; factor 2: 0.40). One possible explanation is that the item has a generic formulation in terms of who these “others” may be. Alternatively, this may reflect cultural issues (e.g., the phenomenon of ego dissolution in Brazil might involve a feeling of union with the universe and nature, but not with other people). Another item that merits attention is #11 (“I felt far less absorbed with my own issues and concerns”), since it surprisingly loaded onto the ego inflation factor. This item also has a grammatical construction that could be problematic, since it involves the adverbial intensifier “far less,” which could force more conservative responses. Furthermore, “issues and concerns” are very diverse mental events, which makes understanding of the item very open to interpretation.

As an alternative to the two-factor model, a three-factor structure was proposed by the Hull Method and the Kaiser-Guttman criterion. In this model, items coalesce around aspects related to self-confidence and assertiveness (items 2, 3, 5, 8, 10, 11, 14, and 16), ego dissolution (items 1, 3, 7, 9, 13, and 15), and ego inflation (items 4, 6, and 12). Comparisons between different substances showed that users of classical psychedelics scored higher for factor 1 than users of cannabis and alcohol but scored lower compared to cocaine and MDMA users for factor 3. For the ego dissolution factor (factor 2), users of classic psychedelics scored higher than other users, except for LSD in relation to MDMA and cocaine, although there were no statistically significant differences. This suggests that there may be cognitive empowerment during the psychedelic experience that does not necessarily imply increased egocentrism or ego inflation. These results indicate a split within the ego inflation items, with some ego dissolution items also loading onto other factors. This means that there may be different possible arrangements between these three domains of the self, in addition to the simple dissolution versus inflation dichotomy. This is represented, for instance, by item 3 (“I felt at one with the universe”), which loaded onto both the self-confidence/assertiveness and ego dissolution factors. Perhaps this shows that “feeling at one with the universe” can represent not only a feeling of ego loss, but also a sense of cognitive empowerment due to the meaning attributed to the feeling of unity. Further qualitative research may shed light on the specific meaning that users of psychedelics attribute to these concepts. In addition, semantic adaptation studies may also be recommended for items 3, 5, 11, and 13.

A three-factor model was also found in a study by Dworatzyk et al.^33^ that sought to validate the EDI in a German sample. Similarly, these authors also found a factor structure in which the more specific items about ego dissolution loaded onto a factor of their own (except for #6 “I felt at one with the universe,” which also loaded onto this factor in our study, but did not in the study by Dworatzyk et al.).^33^ In addition, the authors found a second factor with six items referring to aspects of ego inflation and a third factor with the items “I felt at one with the universe,” “I felt a sense of union with others,” “I felt far less absorbed by my own issues and concerns,” and “I felt particularly sure-of-myself.” The last two of these items also had cross-loadings onto the ego inflation factor.^33^

In our analyses, factors 1 and 2 of the three-factor solution were positively correlated with nature relatedness, although the correlations were weak. Recent data from an online survey show that there is a positive association between classic psychedelics and nature relatedness in Brazil,^21^ but the role the ego dissolution phenomenon plays in this associations remains unclear. Other studies exploring this topic found that nature relatedness in psychedelic experience correlates with the extent of ego dissolution but depends on the perceived influence of natural surroundings during the acute psychedelic state.^19^ Our findings may suggest that nature relatedness has a weaker association in Brazil in relation to other world regions. Conversely, sampling for the current study focused primarily on an urban setting, and this may have impacted results. Future studies expanding recruitment to non-urban settings (e.g., the Amazon region) may be important to better clarify this topic. Nevertheless, given that the current study is observational, direction of causality cannot be ascertained, so it is possible that participants with higher nature relatedness present more intense ego dissolution for other reasons.

Also, none of the scale’s factors had a statistically significant correlation with the life satisfaction scale. Given that other studies have demonstrated positive associations between ego dissolution and life satisfaction,^7,24^ including a study that used the same measurement scales (EDI and SWLS),^25^ it is possible that this reflects cultural issues or sampling biases. Life satisfaction is a construct that greatly varies and is influenced by different factors across cultures.^34,35^ To the best of our knowledge, there are no other data on life satisfaction and psychedelic use in Brazil, so future studies should explore this issue further.

Regarding limitations, some cultural difficulties of translating the term “ego,” as well as the semantic formulation of some items, may have generated conflict in the answers. Additionally, the study sample was predominantly recruited from college students, which biased its composition towards white ethnicity and higher educational achievement. It is possible, however, that this group is more represented among users of psychedelics. In any case, it is important to conduct new studies with more diverse samples, perhaps also avoiding biases linked to online data collection.

## Conclusion

This is the first study to adapt and validate the EDI in a developing country. Considering the recent explosion of studies with psychedelics, the existence of an instrument duly validated and adapted to evaluate such a central aspect of the psychedelic experience is very relevant. The EDI-BR showed acceptable psychometric properties and further validation should be pursued, including determination of the instrument’s predictive validity. Considering the internal consistency and fit indexes of the two different factor solutions, as well as the initial evidence of discriminant validity provided by comparing scores between users of different substances, we recommend using the scale with 16 dissolution/inflation items in Brazil and the three-factor solution model for calculating scores, until further studies on the validity of the EDI-BR are available.
