# Supplementary material for: Adaptation and latent structure of the Brazilian version of the Ego Dissolution Inventory (EDI-BR): an exploratory study
Source: Trends Psychiatry Psychother. 2024 Apr 23;46:e20220491. doi: 10.47626/2237-6089-2022-0491 (PMC11332685; doi:10.47626/2237-6089-2022-0491)
Supplement: Supplementary file 1 [file 2238-0019-trends-46-e20220491-suppl.pdf]

http://doi.org/10.47626/2237-6089-2022-0491

Table S1 - Comparisons between substances for EDI factor 1

**Substance**	**Mean (SD)**	**∆M (95%CI)**	**Sig**
Magic mushrooms<br>DMT	294.03 (191.29)<br>337.11 (200.43)	-43.07 (-134.88-48.73)	> 0.999
Magic mushrooms<br>LSD	294.03 (191.29)<br>255.43 (169.46)	38.60 (-23.93-101.13)	> 0.999
Magic mushrooms<br>MDMA	294.03 (191.29)<br>360.40 (189.51)	-66.36 (-154.15-21.43)	0.449
Magic mushrooms<br>Cocaine	294.03 (191.29)<br>294.69 (199.53)	-.65 (-144.74-143.43)	> 0.999
Magic mushrooms<br>Cannabis	294.03 (191.29)<br>130.58 (164.63)	163,45 (68.48-258.43)	< 0.001*
Magic mushrooms<br>Alcohol	294.03 (191.29)<br>157.56 (150.28)	136.47 (53.47-219.46)	< 0.001*
DMT<br>LSD	337.11 (200.43)<br>255.43 (169.46)	81.67 (-8.94-172.29)	0.129
DMT<br>MDMA	337.11 (200.43)<br>360.40 (189.51)	-23.29 (-132.88-86.30)	> 0.999
DMT<br>Cocaine	337.11 (200.43)<br>294.69 (199.53)	42.42 (-115.89-200.73)	> 0.999
DMT<br>Cannabis	337.11 (200.43)<br>130.58 (164.63)	206.52 (91.10-321.95)	< 0.001*
DMT<br>Alcohol	337.11 (200.43)<br>157.56 (150.28)	179.54 (73.76-285.32)	< 0.001*
LSD<br>MDMA	255.43 (169.46)<br>360.40 (189.51)	-104.96 (-191.51--18.41)	0.005*
LSD<br>Cocaine	255.43 (169.46)<br>294.69 (199.53)	-39.25 (-182.59-104.07)	> 0.999
LSD<br>Cannabis	255.43 (169.46)<br>130.58 (164.63)	124.85 (31.02-218.68)	0.001*
LSD<br>Alcohol	255.43 (169.46)<br>157.56 (150.28)	97.87 (16.19-179.55)	0.006*
MDMA<br>Cocaine	360.40 (189.51)<br>294.69 (199.53)	65.71 (-90.31-221.73)	> 0.999
MDMA<br>Cannabis	360.40 (189.51)<br>130.58 (164.63)	229.81 (117.56-342.07)	< 0.001*
MDMA<br>Alcohol	360.40 (189.51)<br>157.56 (150.28)	202.83 (100.51-305.15)	< 0.001*
Cocaine<br>Cannabis	294.69 (199.53)<br>130.58 (164.63)	164.11 (3.94-324.27)	0.039*
Cocaine<br>Alcohol	294.69 (199.53)<br>157.56 (150.28)	137.12 (-16.25-290.49)	0.138
Cannabis<br>Alcohol	130.58 (164.63)<br>157.56 (150.28)	-26.98 (-81.56-135.53)	> 0.999

95%CI = 95% confidence interval; EDI = Ego Dissolution Inventory; SD = standard deviation.

http://doi.org/10.47626/2237-6089-2022-0491

Table S2 - Comparisons between substances for EDI factor 2

Substance	Mean (SD)	∆M (95%CI)	Sig
Magic mushrooms<br>DMT	241.66 (166.72)<br>329.74 (158.87)	-88.08 (-162.13--14.04)	0.006*
Magic mushrooms<br>LSD	241.66 (166.72)<br>179.08 (147.17)	62.58 (12.15-113.01)	0.004*
Magic mushrooms<br>MDMA	241.66 (166.72)<br>123.17 (133.72)	118.49 (47.68-189.30)	< 0.001*
Magic mushrooms<br>Cocaine	241.66 (166.72)<br>99.50 (122.31)	142.16 (25.95-258.37)	0.004*
Magic mushrooms<br>Cannabis	241.66 (166.72)<br>88.70 (118.14)	152.96 (76.36-229.56)	< 0.001*
Magic mushrooms<br>Alcohol	241.66 (166.72)<br>54.10 (89.22)	187.56 (120.63-254.50)	< 0.001*
DMT<br>LSD	329.74 (158.87)<br>179.08 (147.17)	150.66 (77.58-223.75)	< 0.001*
DMT<br>MDMA	329.74 (158.87)<br>123.17 (133.72)	206.57 (118.19-294.96)	< 0.001*
DMT<br>Cocaine	329.74 (158.87)<br>99.50 (122.31)	230.24 (102.56-357.93)	< 0.001*
DMT<br>Cannabis	329.74 (158.87)<br>88.70 (118.14)	241.05 (147.96-334.14)	< 0.001*
DMT<br>Alcohol	329.74 (158.87)<br>54.10 (89.22)	275.65 (190.33-360.96)	< 0.001*
LSD<br>MDMA	179.08 (147.17)<br>123.17 (133.72)	55.91 (-13.89-125.72)	0.311
LSD<br>Cocaine	179.08 (147.17)<br>99.50 (122.31)	79.58 (-36.02-195.18)	0.757
LSD<br>Cannabis	179.08 (147.17)<br>88.70 (118.14)	90.38 (14.71-166.06)	0.006*
LSD<br>Alcohol	179.08 (147.17)<br>54.10 (89.22)	124.98 (59.11-190.86)	< 0.001*
MDMA<br>Cocaine	123.17 (133.72)<br>99.50 (122.31)	23.67 (-102.16-149.50)	> 0.999
MDMA<br>Cannabis	123.17 (133.72)<br>88.70 (118.14)	34.47 (-56.07-125.01)	> 0.999
MDMA<br>Alcohol	123.17 (133.72)<br>54.10 (89.22)	69.07 (-13.45-151.60)	0.229
Cocaine<br>Cannabis	99.50 (122.31)<br>88.70 (118.14)	10.80 (-118.38-139.98)	> 0.999
Cocaine<br>Alcohol	99.50 (122.31)<br>54.10 (89.22)	45.40 (-78.29-169.10)	> 0.999
Cannabis<br>Alcohol	88.70 (118.14)<br>54.10 (89.22)	34.60 (-52.94-122.15)	> 0.999

95%CI = 95% confidence interval; EDI = Ego Dissolution Inventory; SD = standard deviation.

http://doi.org/10.47626/2237-6089-2022-0491

Table S3 - Comparisons between substances for EDI factor 3

**Substance**	**Mean (SD)**	**∆M (95%CI)**	**Sig**
Magic mushrooms<br>DMT	38.41 (53.55)<br>31.06 (48.00)	7.34 (-23.14-37.84)	> 0.999
Magic mushrooms<br>LSD	38.41 (53.55)<br>55.09 (61.37)	-16.67 (-37.47-4.13)	0.309
Magic mushrooms<br>MDMA	38.41 (53.55)<br>70.77 (79.74)	-32.36 (-61.52--3.20)	0.016*
Magic mushrooms<br>Cocaine	38.41 (53.55)<br>102.69 (83.54)	-64.27 (-112.13--16.41)	0.001*
Magic mushrooms<br>Cannabis	38.41 (53.55)<br>24.58 (46.30)	13.83 (-17.72-45.38)	> 0.999
Magic mushrooms<br>Alcohol	38.41 (53.55)<br>38.81 (56.45)	-.39 (-27.96-27.18)	> 0.999
DMT<br>LSD	31.06 (48.00)<br>55.09 (61.37)	-24.02 (-54.14-6.10)	0.320
DMT<br>MDMA	31.06 (48.00)<br>70.77 (79.74)	-39.71 (-76.11--3.31)	0.019*
DMT<br>Cocaine	31.06 (48.00)<br>102.69 (83.54)	-71.62 (-124.21--19.04)	< 0.001*
DMT<br>Cannabis	31.06 (48.00)<br>24.58 (46.30)	6.48 (-31.86-44.82)	> 0.999
DMT<br>Alcohol	31.06 (48.00)<br>38.81 (56.45)	-7.74 (-42.88-27.40)	> 0.999
LSD<br>MDMA	55.09 (61.37)<br>70.77 (79.74)	-15.69 (-44.46-13.08)	> 0.999
LSD<br>Cocaine	55.09 (61.37)<br>102.69 (83.54)	-47.60 (-95.22-.02)	0.050*
LSD<br>Cannabis	55.09 (61.37)<br>24.58 (46.30)	30.50 (-.68-61.69)	0.062
LSD<br>Alcohol	55.09 (61.37)<br>38.81 (56.45)	16.28 (-10.87-43.44)	> 0.999
MDMA<br>Cocaine	70.77 (79.74)<br>102.69 (83.54)	-31.91 (-83.74-19.91)	> 0.999
MDMA<br>Cannabis	70.77 (79.74)<br>24.58 (46.30)	46.19 (8.90-83.48)	0.004*
MDMA<br>Alcohol	70.77 (79.74)<br>38.81 (56.45)	31.97 (-2.02-65.95)	0.089
Cocaine<br>Cannabis	102.69 (83.54)<br>24.58 (46.30)	78.11 (24.90-131.31)	< 0.001*
Cocaine<br>Alcohol	102.69 (83.54)<br>38.81 (56.45)	63.88 (12.94-114.83)	0.003*
Cannabis<br>Alcohol	24.58 (46.30)<br>38.81 (56.45)	-14.22 (-50.28-21.83)	> 0.999

95%CI = 95% confidence interval; EDI = Ego Dissolution Inventory; SD = standard deviation.

http://doi.org/10.47626/2237-6089-2022-0491

Supplementary Material S4 - Brazilian version of the Ego Dissolution Inventory (EDI) with instructions

**INVENTÁRIO DE DISSOLUÇÃO DO EGO**	
**Instruções:**	
Por favor, pense em uma experiência marcante que você teve com a substância e avalie a intensidade em que cada afirmação se aplica para a experiência em questão.	
Abaixo de cada afirmação tem uma linha com as extremidades "**Não** mais que usualmente" e "**Sim**, experienciei isso completamente/inteiramente". A linha é utilizada para marcar as alterações em relação ao seu estado normal. Seu estado normal corresponde à marca na extremidade esquerda da escala, isto é "Não mais que usualmente".	
Apenas marque no extremo da escala se isso **<u>verdadeiramente</u>** for o caso.	
**Itens**	
1.	Experienciei uma dissolução do meu "Eu" ou ego.
2.	Eu me senti particularmente assertivo.
3.	Eu me senti um com o universo.
4.	Eu me senti mais importante ou especial do que os outros.
5.	Eu senti união com os outros.
6.	Eu senti meu "eu" inflado.
7.	Eu experienciei uma diminuição no meu sentido de auto importância.
8.	Eu me senti particularmente certo de mim mesmo.
9.	Eu experienciei uma desintegração do meu "Eu" ou ego.
10.	Eu me senti especialmente motivado e competitivo.
11.	Eu me senti muito menos absorvido com minhas próprias questões e preocupações.
12.	Eu senti como se o meu ponto de vista valesse mais do que o de outras pessoas.
13.	Perdi toda a sensação de ego.
14.	Eu me senti particularmente autoconfiante.
15.	Toda noção de eu e identidade se dissolveu.
16.	Eu me senti particularmente seguro.
